# P-1303. Moving Research into Action: The Use of Hospital Admission Data as a Measure of Ukrainian Child Migrants’ and War Refugees’ Health Needs in the First 100 Days of the War

**DOI:** 10.1093/ofid/ofae631.1484

**Published:** 2025-01-29

**Authors:** Katarzyna Lewtak, Dorota Kleszczewska, Anna Dzielska, Joanna Mazur, Agnieszka Sochoń-Latuszek, Katarzyna Kukuła

**Affiliations:** Medical University of Warsaw, Warsaw, Mazowieckie, Poland; Institute of Mother and Child Foundation, Warsaw, Poland, Warsaw, Mazowieckie, Poland; Institute of Mother and Child, Warsaw, Mazowieckie, Poland; University of Zielona Góra, Collegium Medicum, Zielona Gora, Lubuskie, Poland; UNICEF Refugee Response Office in Poland, Warsaw, Poland, Warsaw, Mazowieckie, Poland; UNICEF Refugee Response Office in Poland, Warsaw, Poland, Warsaw, Mazowieckie, Poland

## Abstract

**Background:**

Since the start of the war in Ukraine on February 24th, 2022, Poland has been one of the countries with the largest arrival of migrant and war refugees from Ukraine. As the presence of Ukrainian migrant and war refugee children has increased in Poland, studies of their health condition and the use of health services have become of key importance in fulfilling their unmet health needs.

**Methods:**

Data on the hospitalization of Ukrainian pediatric patients in Polish hospitals come from the Nationwide General Hospital Morbidity Study (NGHMS). The analyses were based on the diagnoses and conditions most responsible for the patient’s stay in hospital and coded in accordance with ICD-10.

**Results:**

According to the NGHMS, during the first 100 days of war 2,544 Ukrainian children and adolescents under 18 years of age were admitted to Polish hospitals.

The most frequently reported hospital events among the Ukrainian migrant children were infectious and parasitic diseases (A00-B99) – 24.1%, mainly viral and other specified intestinal infections (A08; 49.3%), other gastroenteritis and colitis of infectious and unspecified origin (A09; 36.2%). Symptoms, signs and abnormal clinical and lab findings (R00-R99 – 17.8%) were the second most frequently reported cause of hospitalization, the majority of which were related to digestive system symptoms - mostly nausea and vomiting and abdominal pain, as well as fever of unknown origin. Diseases of the respiratory system (J00-J99) ranked as the third leading cause of pediatric hospitalization (12.2%), with a predominance of acute bronchitis (J20) and influenza due to identified seasonal influenza virus (J10). 88 Ukrainian children were admitted to hospital due to COVID-19. One third of analyzed hospitalizations (33.1%) had a principal diagnosis of vaccine-preventable disease (VPD).

**Conclusion:**

Our findings suggest a greater focus on preventive healthcare and public health interventions, especially in the area of VPD. As vaccination should be considered a health equity intervention, the tailored vaccination program SAY YES TO VACCINATION in collaboration with UNICEF (SBC Office) has been implemented to increase mothers’ vaccination literacy and address gaps in vaccination coverage among Ukrainian children in Poland.

**Disclosures:**

**All Authors**: No reported disclosures

